# Fever and Confusion in an Elderly Man with AKI

**DOI:** 10.34067/KID.0000000000000193

**Published:** 2023-10-26

**Authors:** Swetha R. Kanduri, Nicholas Carbajal, Juan Carlos Q. Velez

**Affiliations:** 1 Ochsner Clinical School/The University of Queensland, New Orleans, Louisiana; 2 Department of Nephrology, Ochsner Health, New Orleans, Louisiana

**Keywords:** AKI, nephrolithiasis, hydronephrosis, obstructive uropathy, acute kidney failure, acute renal failure, kidney stones, renal function

## Abstract

**Figure:**
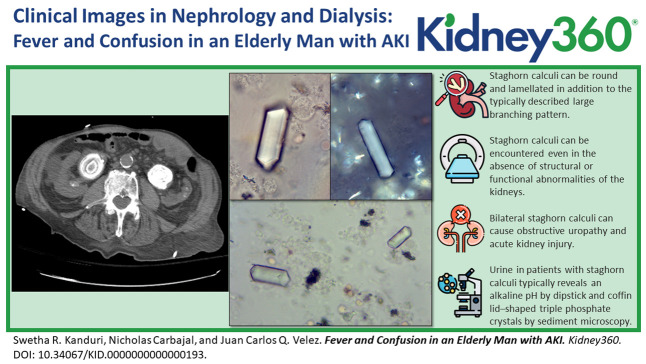

## Case Description

**Figure 1.**
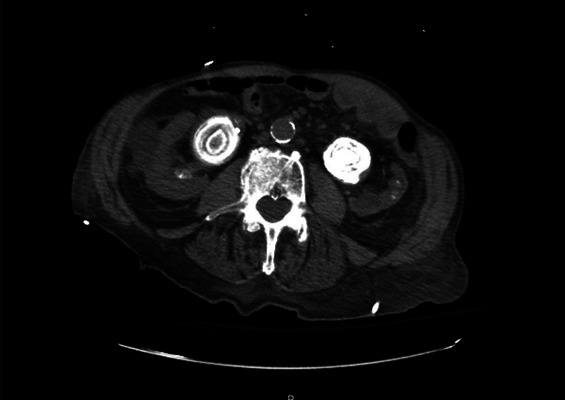
**Noncontrast computed tomography coronal images demonstrate moderate left hydronephrosis from a large lamellated calculus within the renal pelvis measuring 5.0 cm.** Moderate-to-severe right hydronephrosis secondary to large lamellated calculus within the right renal pelvis measuring 5.7 cm and an additional calculus within the proximal aspect of the right ureter measuring 1.8 cm.

**Figure 2.**
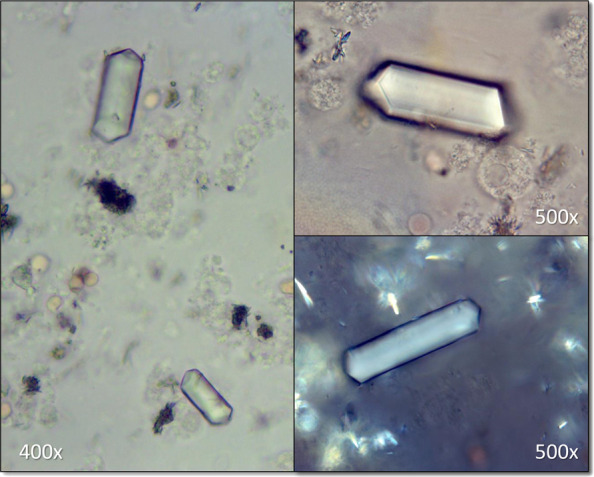
**Representative images of struvite crystals identified by urinary sediment microscopy by bright field illumination.** Bottom right image shows a struvite crystal under partial polarization. Magnification annotated (400–500×).

A 80-year-old man was brought to the emergency department with a 2-day history of fever and confusion. Laboratory values revealed a serum creatinine of 4.2 mg/dL (baseline 1.0 mg/dL). Urinalysis was positive for bacteria and leukocytes. A computed tomography of the abdomen and pelvis reported severe right hydronephrosis and a moderate left hydronephrosis with a 5.7-cm and 5-cm large round lamellated calculi in the right and left renal pelvis, respectively, and were identified as staghorn calculi (Figure 1). Urology and interventional radiology were consulted, and bilateral nephrostomy tubes were placed. Kidney function improved with serum creatinine decreasing to 1.5 mg/dL. Surgical intervention for removal of staghorn calculi was suggested, but the patient opted for a conservative approach. Three weeks later, the patient was readmitted with worsening confusion, hematuria, decreased urine output and elevated serum creatinine of 2.8 mg/dL. The patient developed septic shock with blood and urine cultures positive for *Proteus mirabilis*. Repeat computed tomography scanning revealed mildly improved bilateral hydronephrosis and persistent right and left renal pelvic calculi. Urinary sediment microscopy showed coffin lid–shaped struvite crystals (Figure 2). Nephrostomy tubes were exchanged with minimal improvement in urine output. Unfortunately, the patient opted to pursue comfort measures and refused further interventions.

## Discussion

Staghorn calculi, also referred to as struvite or triple phosphate (Mg-NH_4_-P0_4_), are complex kidney stones that account for 4% of urinary stones. They are predominantly seen in women and are associated with infection in 50%–70% of the cases.^1^ Urease generated by bacteria, (*Proteus*, *Klebsiella*, or *Pseudomonas*) hydrolyzes urea to ammonia and increases the urine pH. Alkaline pH favors the precipitation of ammonium (NH_4_^+^), magnesium (Mg^2+^), and phosphate (PO_4_^3−^) ions to form triple phosphate crystals.

Staghorn calculi are large branching stones that completely or partially pervade the pelvic calyceal system. Bilateral staghorn calculi leading to hydronephrosis are rare and are generally encountered in patients with congenital abnormalities of the kidneys^2^ or with neurogenic bladder.^3^ In a report of a young patient with tetraplegia and indwelling catheter, recurrent bilateral staghorn calculi were noted requiring multiple surgical interventions. The patient described herein is unique in that the staghorn are round and lamellated in appearance in contrast to the typically described branching pattern, and the patient had no preexisting structural abnormalities.

Although the majority of staghorn calculi are associated with an infection, they have also been reported to be composed of metabolic stones, comprising calcium phosphate, calcium oxalate, and uric acid.^4^ The exact mechanism for the metabolic shift is not well understood and may be partly attributed to dietary trends. Percutaneous nephrolithotomy is considered gold standard for the management of staghorn calculi.

## Teaching Points

- Staghorn calculi can be round and lamellated in addition to the typically described large branching pattern.
- Staghorn calculi can be encountered even in the absence of structural or functional abnormalities of the kidneys.
- Bilateral staghorn calculi can cause obstructive uropathy and acute kidney injury.
- Urine in patients with staghorn calculi typically reveals an alkaline pH by dipstick and coffin lid–shaped triple phosphate crystals by sediment microscopy.
